# Metformin attenuates myocardial ischemia-reperfusion injury via up-regulation of antioxidant enzymes

**DOI:** 10.1371/journal.pone.0182777

**Published:** 2017-08-17

**Authors:** Xiaoling Wang, Lei Yang, Licheng Kang, Jing Li, Liang Yang, Jincai Zhang, Jie Liu, Mengmeng Zhu, Qiong Zhang, Yanna Shen, Zhi Qi

**Affiliations:** 1 Department of Histology and Embryology, School of Medicine, Nankai University, Tianjin, China; 2 Tianjin Institute of Acute Abdominal Diseases of Integrated Traditional Chinese and Western Medicine, Tianjin Nankai Hospital, Tianjin, China; 3 Department of Microbiology, School of Laboratory Medicine, Tianjin Medical University, Tianjin, China; 4 National Clinical Research Center of Kidney Diseases, Beijing, China; Temple University, UNITED STATES

## Abstract

The objective was to examine the protective effect of metformin (Met) on myocardial ischemia-reperfusion (IR) injury and whether the mechanism was related to the AMPK/ antioxidant enzymes signaling pathway. Rat Langendorff test and H_2_O_2_-treated rat cardiomyocytes (H9c2) were used in this study. Met treatment significantly improved left ventricular (LV) function, reduced infarct size and CK-MB release in comparison with IR group. Decreased TUNEL staining positive cells were also observed in IR+Met group *ex vivo*. Met treatment markedly inhibited IR inducing cell death and significantly decreased apoptosis with few generations of reactive oxygen species (ROS) in H9c2 cells in comparison with IR group. Up-regulated expressions of phosphorylated LKB1/AMPK/ACC, as well as down-regulated expressions of apoptotic proteins (Bax and cleaved caspase 3) were found in IR+Met group when compared to the IR group. Importantly, Met significantly up-regulated the expression of antioxidant enzymes (MnSOD and catalase) during IR procedure either *ex vivo* or *in vitro*. Compound C, a conventional inhibitor of AMPK, abolished the promoting effect of Met on antioxidant enzymes, and then attenuated the protective effect of Met on IR injury *in vitro*. In conclusion, Met exerted protective effect on myocardial IR injury, and this effect was AMPK/ antioxidant enzymes dependent.

## Introduction

Myocardial infarction (MI) is one of the most common cardiovascular diseases with high mortality. Early reperfusion by primary percutaneous coronary intervention (PCI) remains the most effective treatment for MI [1]. However, the consequent reperfusion to the ischemic myocardium can induce a secondary injury, myocardial ischemia-reperfusion (IR) injury [2]. Myocardial IR causes contractile dysfunction, results in cardiomyocyte hypertrophy, myocardial fibrosis and finally leads to cell death of cardiomyocytes [3, 4]. Despite major progress in the treatment of myocardial IR injury during the last few decades, the therapeutic effect has not significantly improved. Therefore, attenuation of myocardial IR injury remains an urgent priority in the treatment for MI.

It is well known that the excessive generation of reactive oxygen species (ROS) resulting in redox imbalance during reperfusion period plays key roles in the pathogenesis of IR injury [5]. Excessive generation of ROS is involved in several detrimental processes, including cell death and apoptosis triggered by the opening of the mitochondrial permeability transition pores (mPTP) [6, 7]. Antioxidant agents can effectively decrease generation of ROS, therefore, antioxidant therapy, especially activation of endogenous antioxidant enzymes, such as catalase and manganese superoxide dismutase (MnSOD) seems to be a potential therapeutic approach for myocardial IR injury [8, 9].

Metformin (Met), an orally administered biguanide drug, is now widely used to lower blood glucose concentration in patients with type 2 diabetes mellitus (T2D). The cardioprotective effect of Met has been documented as well [10–12]. Interestingly, United Kingdom Prospective Diabetes Study (UKPDS) demonstrated that metformin decreased the risk of cardiovascular diseases of T2D patients when compared to other conventional therapies [13], indicating that the cardioprotective effect of MET is not simply attributed to its anti-hyperglycemic effects alone. The molecular mechanisms for the cardioprotective effect of MET are not fully understood. Most studies documented that the MET exerted cardioprotective effect via the activation of AMP activated protein kinase (AMPK) [10,14,15], whereas Xu et. al [16] reported that Met protected against systolic overload-induced heart failure independent of AMPK. In addition, Met has been shown to exhibit antioxidant properties in various disorders [17,18], we wonder whether the protective effect of Met against myocardial IR injury is through the elevation of endogenous antioxidant enzymes.

Therefore, the present study investigated the cardioprotective effect of Met on myocardial IR injury and determined whether this effect was involved in AMPK/ antioxidant enzymes signaling pathway.

## Materials and methods

### Animals

Male Sprague-Dawley rats (10 weeks old) were purchased from the Institute of Laboratory Animal of Chinese Medical Science Academy, Beijing, China. Rats were maintained in a temperature-controlled room (25°C) with a natural day/night cycle and fed with a standard rodent diet and water. This study was carried out in strict accordance with the recommendations in the Guide for the Care and Use of Laboratory Animals of Nankai University. The protocol was approved by the Animal Care Committee of School of Medicine, Nankai University, (Permit Number: 10017). The rats were anesthetized with i.p. injection of 10% chloral hydrate, followed by cervical dislocation prior to removal of the hearts. All efforts were made to minimize suffering.

### Langendorff perfusion

Thirty-two rats were randomly divided into three groups as follows: (1) Control (Con) group (n = 6/9, the former means the amount of rats that successfully performed the Langendorff test in each group and the latter means total number in this group); (2) IR group (n = 8/11); (3) IR +Met group (n = 8/12). Rat hearts were quickly removed from heart cavity and connected to Langendorff apparatus by inserting a perfusion cannula into the aorta. Isolated hearts were stabilized for 30 min with Krebs-Henseleit (K–H) solution, and then subjected to global, no-flow ischemia for 30 min and 60 min reperfusion with a flow speed of 4 ml/min in IR group. No ischemia was subjected to isolated heart in Con group. Met (100μM) was added into K-H solution in IR+Met group 15min prior to the ischemia period. Left ventricular developed pressure (LVDP), left ventricular end diastolic pressure (LVEDP), maximal rate of rise of left ventricular pressure (+dp/dp max) and maximal rate of decrease of left ventricular pressure (-dp/dt max) were recorded by a data acquisition device (RM6240B, Chengdu, China). Perfusion solutions (collected at 10, 70, 90 and 120 min) were collected for further assessments. When the Langendorff test was finished, the heart samples were collected to examine the area of myocardial infarction, apoptosis of myocardial cells and expression levels of related mRNA and proteins.

### Assessment of myocardial infarction

Rat hearts were removed at the end of reperfusion and immediately frozen in -20°C refrigerator for 1h. Then, hearts were incubated in 1% triphenyl tetrazolium chloride (TTC) solution (Solarbio, Beijing, China) at 37°C for 15min, followed by immersion in 4% paraformaldehyde solution for 30min. Five cross-sectional slices were taken and imaged on a digital camera, and the percentage of infarct area (white area) to total ventricular area was calculated using Image J software.

### Measurement of CK-MB release

Perfusion solution samples were collected at 10, 70, 90 and 120 min. The CK-MB levels were measured using a rat CK-MB ELISA kit (Milipore, USA) with a microplate reader (Thermo, China) according to the manufacturer’s instruction.

### TUNEL assay

After Langendorff test, the heart tissues were fixed in paraformaldehyde solution for 24h, embedded in paraffin, and 5 μm sections were obtained. Terminal deoxynucleotidyl transferase-mediated nick end labeling (TUNEL) staining in heart tissues was performed according to the protocol of TUNEL Staining Kit (Roche, Germany) to determine the apoptosis of cardiomyocytes. The apoptotic rate was determined by Image J software.

### Cell culture and treatment

Rat embryonic cardioblasts H9c2 cells were purchased from the Type Culture Collection of the Chinese Academy of Sciences, Shanghai, China. Cells were cultured in the DMEM medium supplemented with 10% heat-inactivated fetal bovine serum (FBS), 100U/mL penicillin, and 100μg/mL streptomycin and incubated at 37°C with 95% air and 5% CO_2_. Once cells reached 60–70% confluence, subsequent experiments were performed. Briefly, H9c2 cells were cultured with 500 µmol/L H_2_O_2_ in DMEM containing 2% FBS for 18 h to mimic IR procedure. Cells were pretreated with Met (50μM), Compound C (an AMPK inhibitor; 10μM), AICAR (an AMPK activator; 100μM) for 24h to confirm the effect on AMPK signaling pathway.

### Immunostaining for cell death and apoptosis

Calcein-AM/PI double staining was performed as described previously [19] to determine the cells survival. Calcein AM positive area rate was degined as the percentage of the calcein AM positive area to the whole cells area. Meanwhile, 1μg/mL Hoechst 33342 (to assess apoptosis; Sigma, USA) and 100nm/L tetramethylrhodamine methyl ester (TMRM, to determine mitochondrial membrane potential; Sigma, USA) were mixed at 37°C for 15min to determine the apoptosis of H9c2 cells. Data were analyzed by Image J software.

### Flow cytometry

Apoptosis was also assessed by flow cytometry. Cells were stained with Annexin V and PI by a FITC Annexin V apoptosis detection kit (BD, USA) as described previously [20]. Apoptotic cells were defined as the Annexin V-FITC positive cells, namely, the sum of the early apoptotic cells (quadrant 3, Q3) and late apoptotic cells (Q2).

### Intracellular ROS measurement

Intracellular ROS level was measured using dichlorofluorescein diacetate (DCFH-DA) staining kit (Invitrogen, USA). In brief, DCFH-DA was diluted by PBS to the final concentration of 10μmol/L and incubated at 37°C for 30min. Then the cells were washed twice with PBS. The images of cells were captured by a fluorescence microscope (Olympus, Japan) and fluorescent intensities were analyzed using Image J software.

### Quantitative real-time PCR (qPCR)

Total RNA in heart tissues and H9c2 cells were isolated with Trizol reagent (Invitrogen, Shanghai, China) as described previously [20]. In brief, 1.0 μg RNA was used and reactions were carried out using the reverse transcription system (Promega, shanghai). Primer sequences were shown as below: MnSOD: 5′-AGGTCGCTTACAGATTGCC-3′ and 5′-CATTCTCCCAGTTGATTACATT-3′. catalase: 5′-CCTCGTTCAAGATGTGGT-3′ and 5′-CACCTTTGCCTTGGAGTA-3′. GAPDH: 5′-TATCGGACGCCTGGTTAC-3′ and 5′-CTGTGCCGTTGAACTTGC-3′. The qPCR was performed using SYBR Green Mix (Promega, Shanghai) in a Bio-Rad IQ5 detection system. The amount of each gene expression was normalized to GAPDH mRNA expression.

### Western blot analysis

Western blot analysis was performed as described previously [19]. In brief, Protein concentration was measured using BCA protein assay Kit (Thermo, USA). The proteins were electroblotted to a polyvinylidene fluoride (PVDF) membrane (Millipore, USA). The membrane was incubated with a primary antibody against phosphorylated(Ser473) and total Akt, phosphorylated (Ser 428) and total LKB1, phosphorylated ACC (Ser79) and total ACC, cleaved caspase-3 (Cell Signaling Technology, USA); AMPK and phosphorylated AMPK (Thr 172), and MnSOD (Abcam, USA); Bax and β-actin(Santa Cruz, CA). Finally, the bands were detected by ECL Kit (Millipore, USA) and quantified by Image J software. The levels of phosphorylated proteins (p-LKB1, p-AMPK, p-ACC and p-Akt) were relative to the total proteins, respectively. And the levels of other proteins (MnSOD, Bax and cleaved caspase-3) were relative to β-actin.

### Statistical analysis

All experiments were repeated three times, and all results were expressed as the mean ± S.E.M. Significant differences between three or more groups were tested by one-way ANOVA followed by multiple comparisons performed with LSD test (SPSS ver. 17). Statistical significance was defined as p < 0.05.

## Results and discussion

### Met attenuated IR-induced LV dysfunction

**Fig 1:**
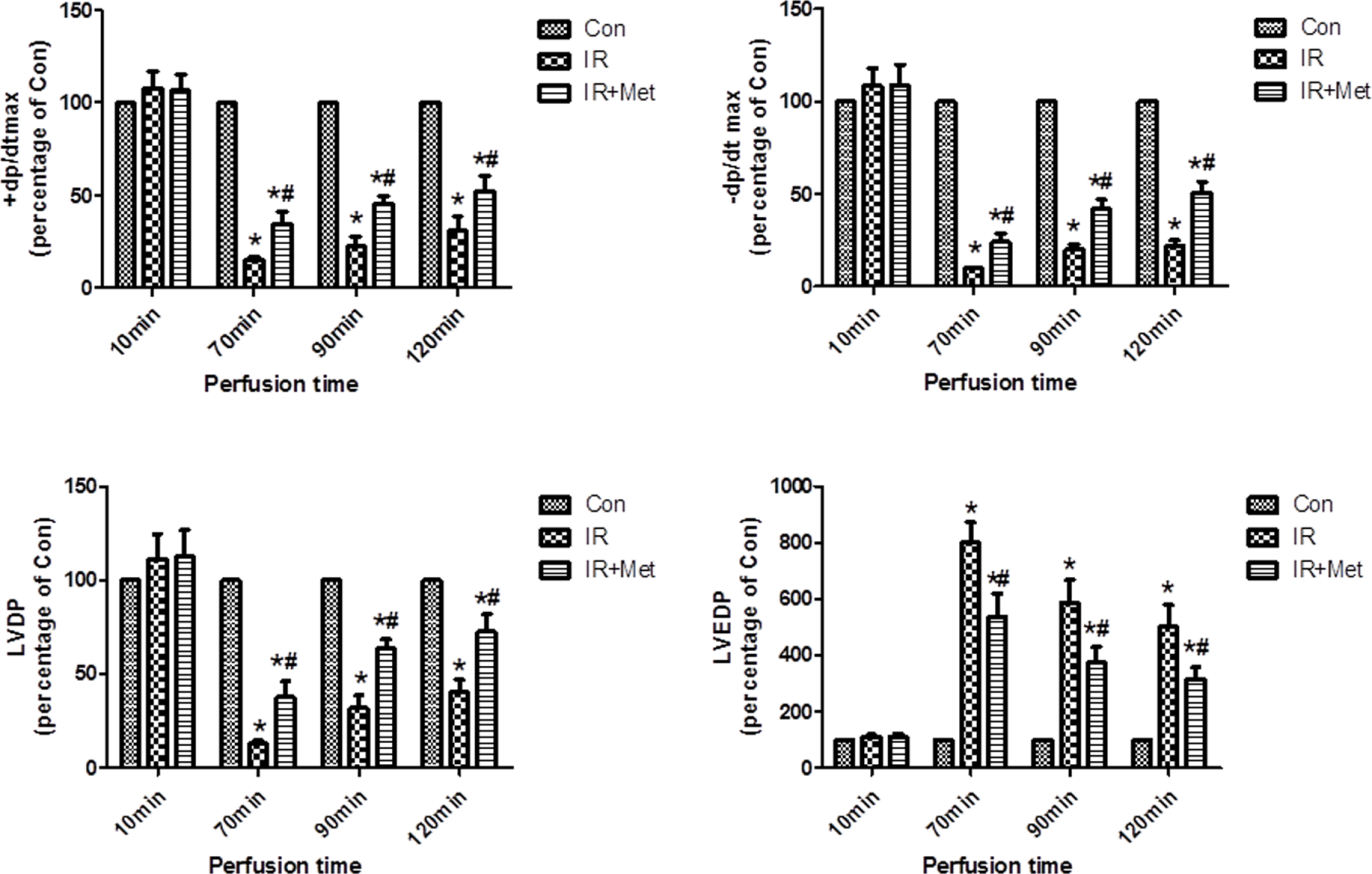
The effect of Met on left ventricular function during myocardial IR. LVDP, LVEDP and ± dp/dt max were recorded and calculated in Langendoff perfusion test. No significant differences were found between 3 groups in stabilization period. Left ventricular function was decreased in reperfusion period whereas Met treatment significantly increased left ventricular function. *p<0.05 vs. Con group, #p<0.05 vs. IR group. n = 6–8 rats/group.

To evaluate the myocardial IR injury, parameters for LV function were recorded and calculated in Langendorff apparatus. No significant differences in the LV function parameters were found between three groups in 10 min of perfusion (stabilization period). IR resulted in decreased ±dp/dtmax, LVDP, and increased LVEDP in comparison with Con group in 70, 90, 120min (reperfusion period), respectively. However, Met treatment significantly improved LV functions in reperfusion period (Fig 1).

### Met reduced heart infarct size and decreased CK-MB release

**Fig 2:**
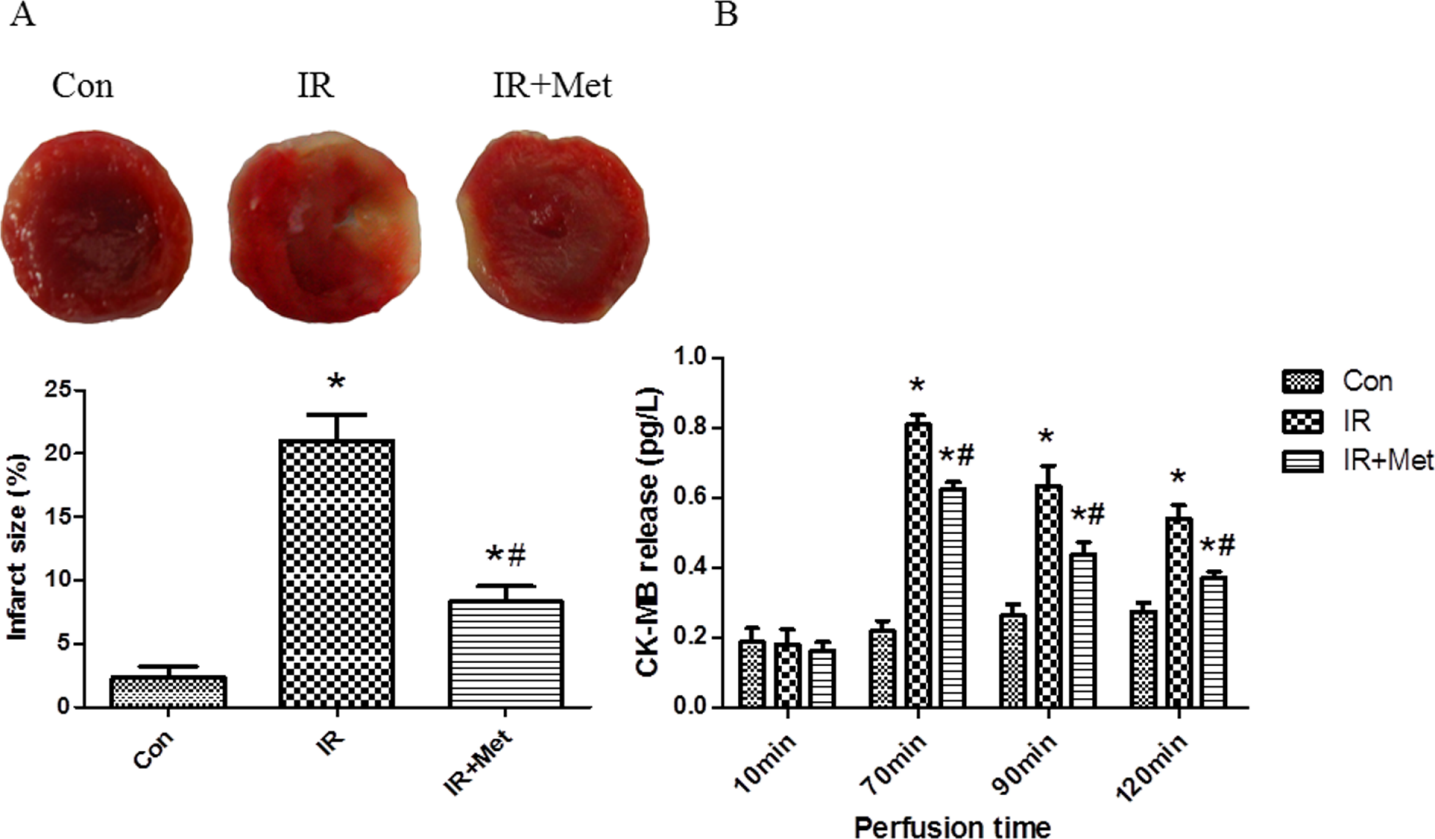
The effect of Met on heart infarct size and CK-MB release. Heart infarct size was evaluated by TTC staining (A). CK-MB release was measured by ELISA (B). *p<0.05 vs. Con group, #p<0.05 vs. IR group.

As shown in Fig 2A, few infarct areas were observed in the Con group. Met treatment significantly decreased infarct size in comparison with IR group. Meanwhile, we found that there were no significant differences between 3 groups in 10min of perfusion (stabilization period), whereas IR group showed increased CK-MB releases in 70, 90, 120min (reperfusion period). Met treatment significantly reduced CK-MB release when compared to the IR group in all three time points during reperfusion period (Fig 2B).

### Met activated AMPK signaling pathways, increased expression of antioxidant enzymes and inhibited apoptosis *ex vivo*

**Fig 3:**
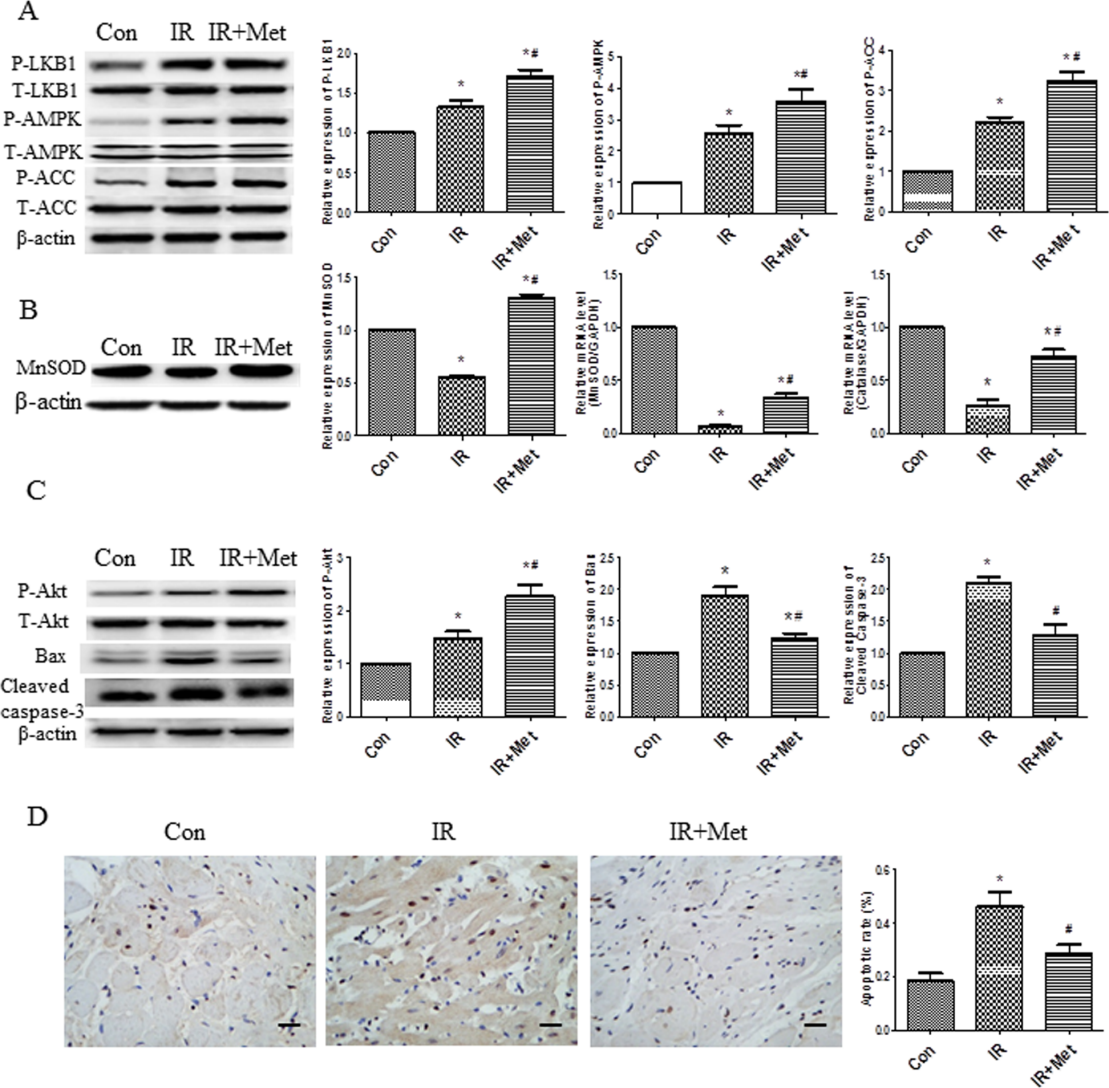
The effect of Met on AMPK signaling pathway, antioxidant enzymes and apoptosis *ex vivo*. The protein levels of LKB1/AMPK/ACC were determined (A). Protein levels of MnSOD, mRNA levels of MnSOD and catalase were determined (B). Akt, Bax, cleaved caspase-3 protein levels were examined (C). TUNEL staining was performed to further confirm apoptosis; apoptotic cells were stained with brown color. The black line represented a scale bar of 50μm (D). *p<0.05 vs. Con group, #p<0.05 vs. IR group.

We detected the expressions of AMPK, phosphorylated LKB1 (up-stream of AMPK) and phosphorylated ACC (down-stream of AMPK) in *ex vivo* study. Met increased the p-LKB1, p-AMPK, p-ACC protein levels in comparison with IR group, indicating that Met treatment activated AMPK signaling pathway (Fig 3A). To determine antioxidant properties of Met during myocardial IR injury, MnSOD and catalase mRNA levels, and protein level of MnSOD were examined. Met treatment significantly up-regulated the protein and mRNA expressions of antioxidant enzymes (Fig 3B). In order to determine cell survival and apoptosis during IR, western blot for Akt, Bax and cleaved caspase-3 were also analyzed. Increased phosphorylated Akt, decreased Bax and cleaved caspase-3 protein levels were observed in IR+Met group when compared to IR group (Fig 3C). Additionally, numerous TUNEL positive cells were found in IR group, whereas Met treatment significantly reduced apoptotic cells in heart tissues (Fig 3D).

### The cardioprotective effect of Met was depended on AMPK signaling pathway

**Fig 4:**
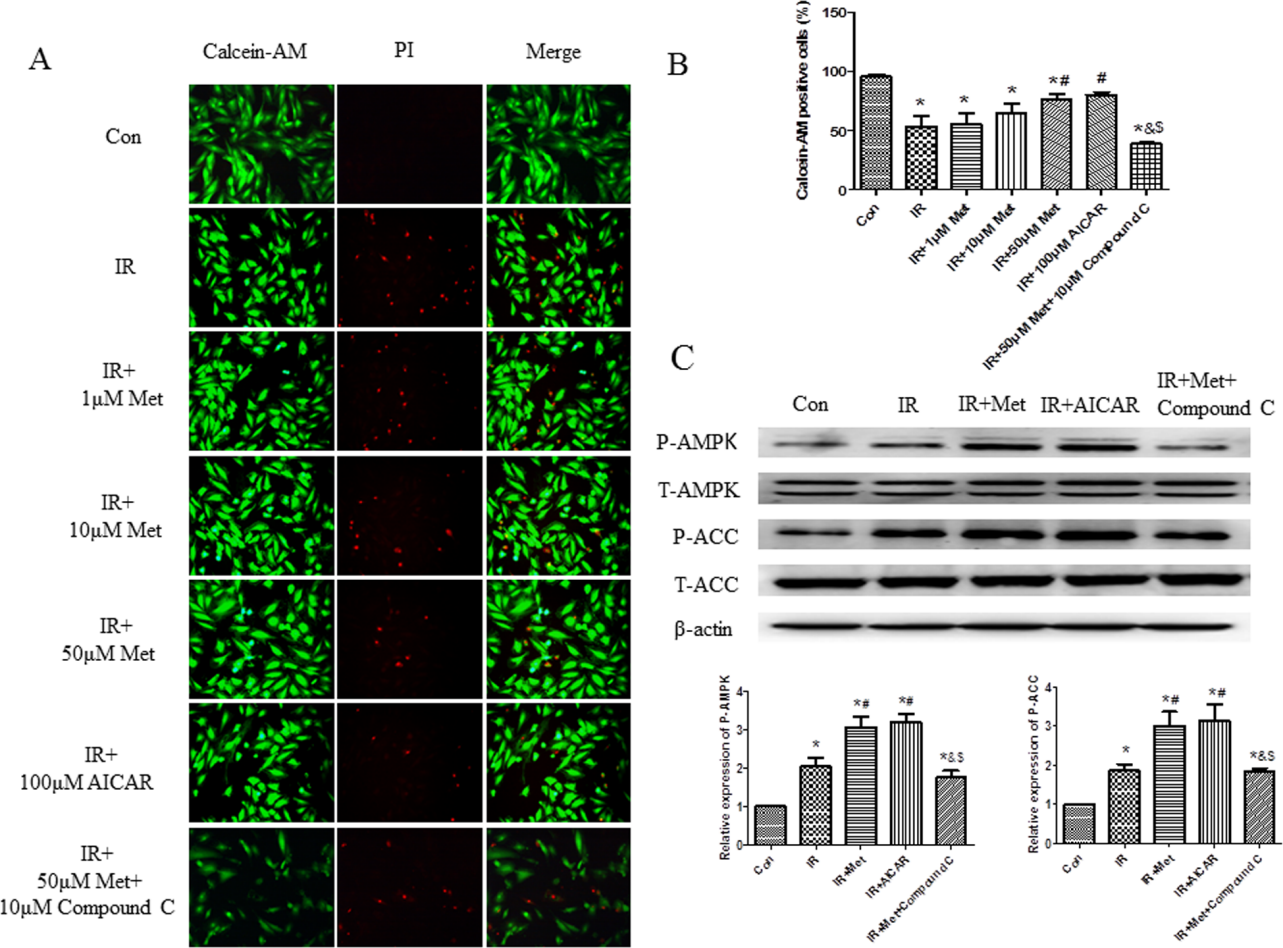
The effect of Met on cell death and AMPK signaling pathway *in vitro*. Calcein AM/PI double staining was performed in H9c2 cells to determine cell death (A). Quantification of survived cells rate was calculated (B). The protein levels of AMPK/ACC were determined in H9c2 cells (C). *p<0.05 vs. Con group, #p<0.05 vs. IR group, & p<0.05 vs. IR+Met group, $ p<0.05 vs. IR+AICAR group.

AICAR (an activator of AMPK) and Compound C (an inhibitor of AMPK) were further used in H9c2 cells IR model to determine whether Met exerted cardioprotective effect via AMPK signaling pathway. We found that 50μM Met treatment markedly inhibited cell death during IR procedure; therefore, the concentration of 50μM of Met was selected to serve as the working concentration in the subsequent studies (Fig 4A and 4B). AICAR treatment group showed similar cardioprotective effect with 50μM Met group during IR, whereas, Compound C treatment abolished the cardioprotective effect of Met, indicating that Met did exert cardioprotective effect via AMPK signaling pathway (Fig 4A–4C).

**Fig 5:**
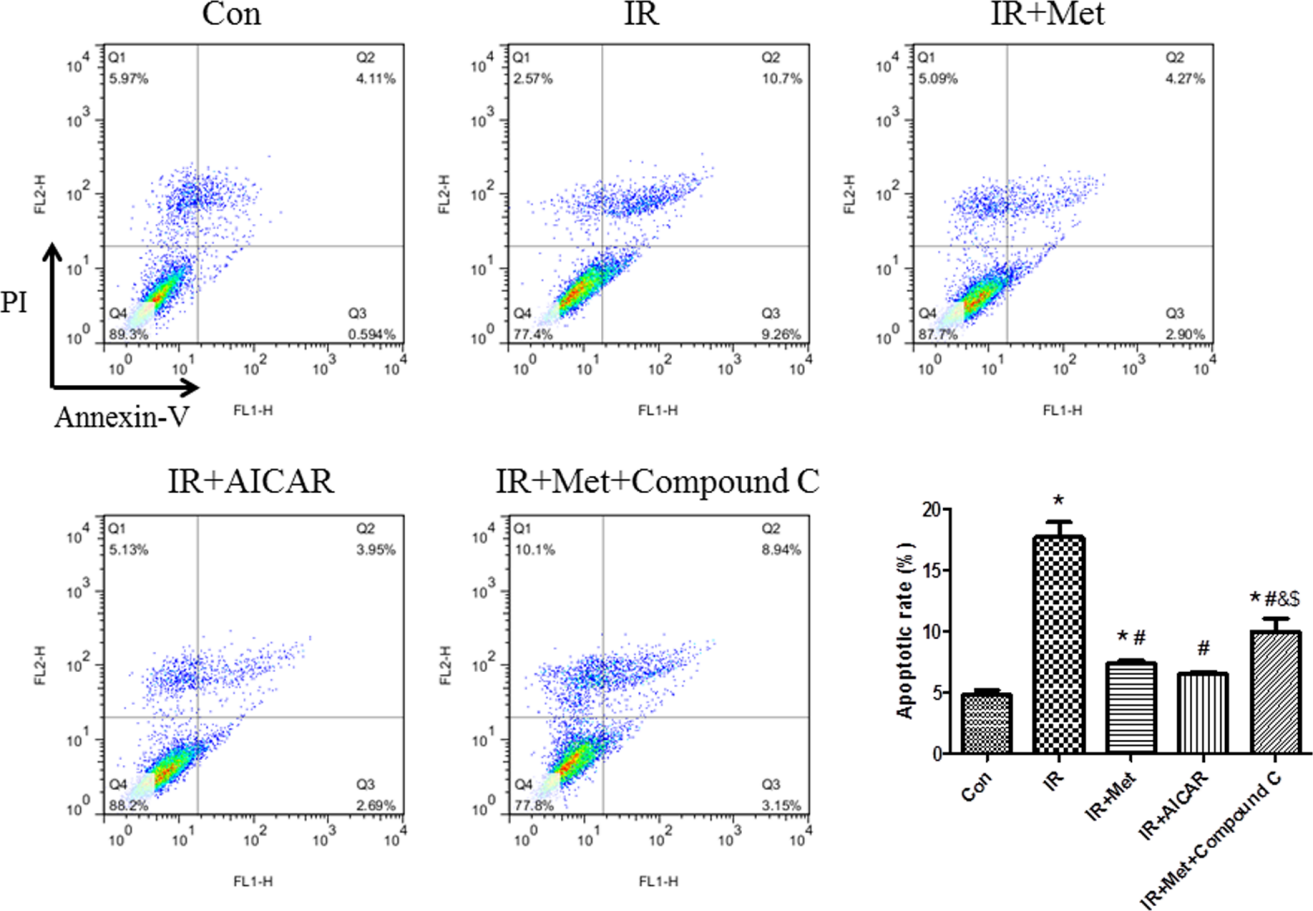
The effect of Met on apoptosis during IR in H9c2 cells. Apoptotic cells were defined as the Annexin V-FITC positive cells (cells in Q2 and Q3). *p<0.05 vs. Con group, #p<0.05 vs. IR group, & p<0.05 vs. IR+Met group, $ p<0.05 vs. IR+AICAR group.

To assess whether Met exerted anti-apoptotic effect via AMPK signaling pathway, flow cytometry test was performed in H9c2 cells. Met and AICAR treatment markedly attenuated IR-induced apoptosis of cardiomyocytes, while Compound C treatment abolished this attenuation by inhibition of AMPK signaling pathway (Fig 5).

**Fig 6:**
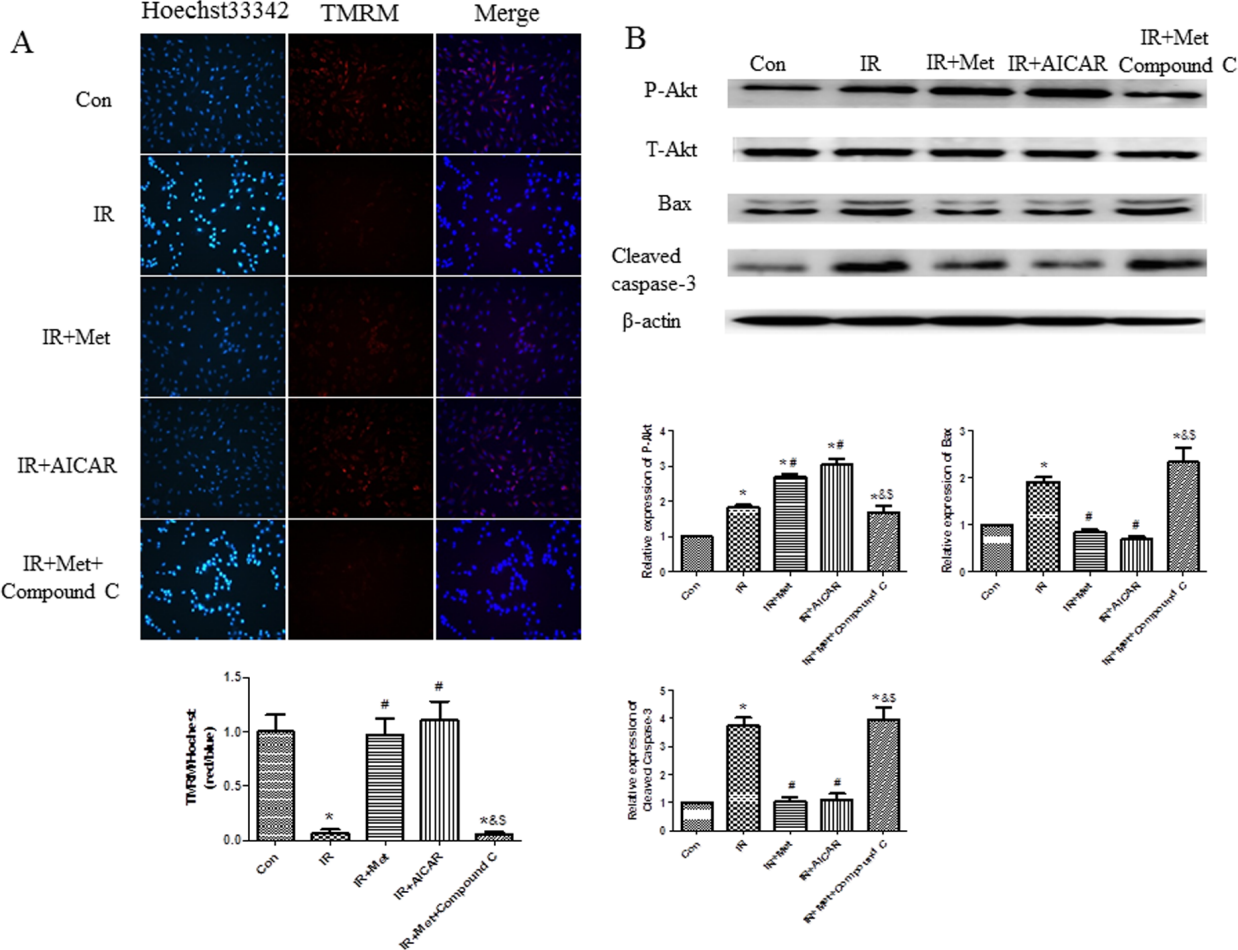
The effect of Met on apoptosis *in vitro*. Hoechst 33342 (for apoptosis) and TMRM (for mitochondrial ΔΨm) double staining was performed (A). Akt, Bax, cleaved caspase-3 protein levels were examined in H9c2 cells (B). *p<0.05 vs. Con group, #p<0.05 vs. IR group, & p<0.05 vs. IR+Met group, $ p<0.05 vs. IR+AICAR group.

It is well known that mitochondrial dysfunction is a major reason for cell apoptosis; therefore, we performed TMRM and Hoechst 33342 double staining to further confirm the anti-apoptotic effect of Met during IR process. Decreased mitochondrial membrane potential (ΔΨm) and increased apoptosis were found in IR group in comparison with Con group. Met treatment increased ΔΨm and reduced apoptosis when compared to the IR group, whereas, Compound C significantly blunted anti-apoptotic effect of Met (Fig 6A). Meanwhile, similar with the results of *ex vivo* study, Met increased phosphorylated Akt, decreased Bax and cleaved caspase-3 protein levels in comparison with IR group. Above mentioned effects of Met were abolished in the presence of Compound C (Fig 6B).

### Met inhibited the generation of ROS during IR procedure

**Fig 7:**
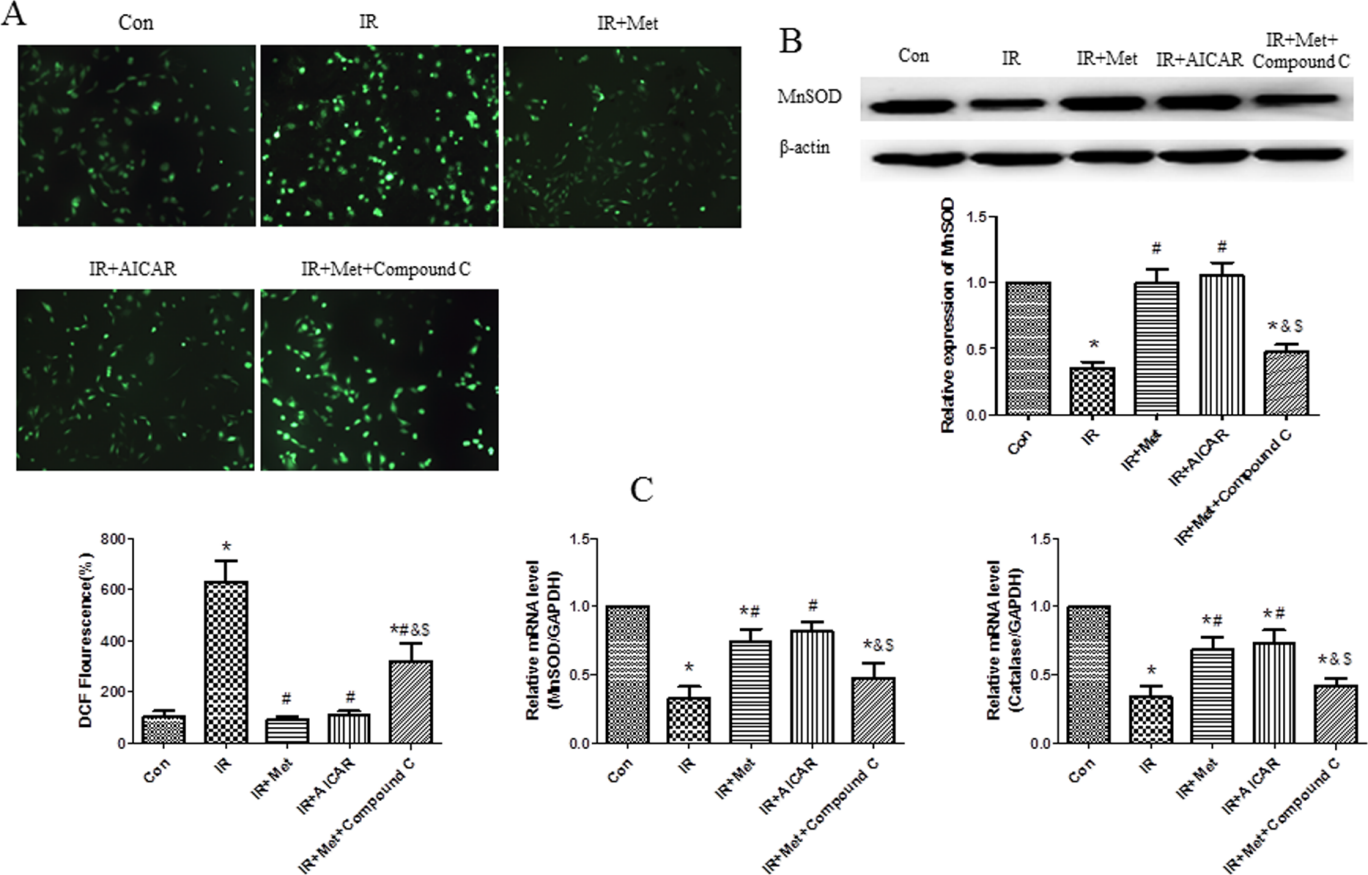
The effect of Met on ROS generation and antioxidant system *in vitro*. DCFH-DA staining was performed to measure intracellular generation of ROS in H9c2 cells (A). MnSOD protein level (B) and mRNA levels of MnSOD and catalase were determined in H9c2 cells (C). *p<0.05 vs. Con group, #p<0.05 vs. IR group, & p<0.05 vs. IR+Met group, $ p<0.05 vs. IR+AICAR group.

DCFH-DA staining was performed to measure intracellular generation of ROS in H9c2 cells. Abundant generations of ROS were observed in the IR group after H_2_O_2_ treatment, while Met obviously lessened the generation of ROS. Combination of Met and Compound C increased the ROS generation when compared to Met alone treatment (Fig 7A). Meanwhile, Met up-regulated endogenous antioxidant system *in vitro*. Met treatment significantly increased MnSOD protein level (Fig 7B), as well as up-regulated mRNA expressions of MnSOD and catalase (Fig 7C) in comparison with IR group, whereas this effect was abolished by the Compound C treatment.

### Schematic model of this study

**Fig 8:**
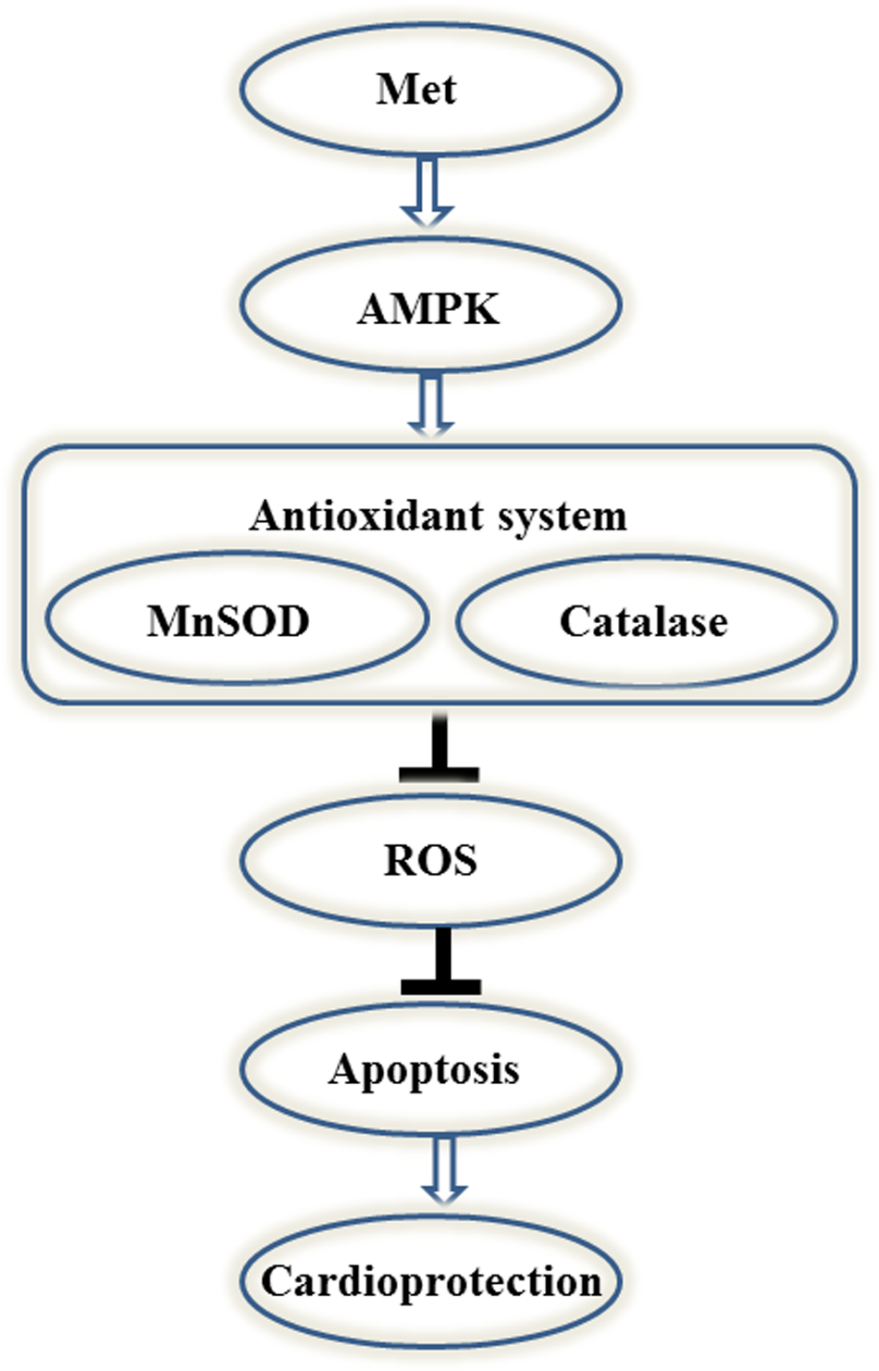
Schematic model of this study. Met protects heart against IR injury through the activation of AMPK/antioxidant enzymes signaling pathway.

Taken together, we can conclude that Met exerted the cardioprotective effect during IR process through the up-regulation of endogenous antioxidant system (eg. MnSOD and catalase), then inhibited the generation of ROS, reduced apoptosis, finally protected myocardiocytes against IR injury. Noticeably, this cardioprotective effect of Met was AMPK dependent (Fig 8).

## Discussion

Metformin, a first line oral anti-diabetic drug for T2D, has been documented for its cardioprotective effect not only in animal studies but also in clinical trials. However, the underlying mechanism for this effect remains controversial. Zhou et al. [21] first reported that Met decreased blood glucose via AMPK signaling pathway. Subsequent studies also demonstrated that Met exerted its various effects, including cardioprotective effect through the activation of AMPK [22–24]. In contrast, recent studies showed that the blood glucose lowering effect, cardioprotective effect and anti-tumor effect of Met are all independent of AMPK [16, 25, 26]. Therefore, it is necessary to confirm whether the cardioprotective effect of Met during IR depends on AMPK signaling pathway.

In current study, Met treatment improved LV function (Fig 1), reduced heart infarct size with decreased CK-MB release (Fig 2), and inhibited apoptosis of cardiomyocytes during IR injury *ex vivo* (Fig 3C and 3D). Significantly activated AMPK signaling pathway was also found in IR+Met group in comparison with IR group (Fig 3A). We then used AICAR and Compound C to further determine the underlying mechanism of Met for the cardioprotective effect on IR *in vitro*. We found that AICAR, an activator of AMPK could mimic the cardioprotective effect of Met during IR process. In contrast, Compound C, an inhibitor of AMPK abolished cardioprotective effect of Met, indicating that Met did protect heart against IR injury via AMPK signaling pathway (Figs 4–6).

Excessive generation of ROS is the key contributor in the development and the progression of myocardial IR injury [6]. Because endogenous antioxidant enzymes, such as MnSOD and catalase can blunt excessive generation of ROS, antioxidant therapy has been identified to attenuate myocardial IR injury. According to the previous studies [15,27], herein, we used H_2_O_2_ to mimic generation of ROS during myocardial IR. We found that H_2_O_2_ treatment caused excessive generation of ROS and numerous dead cells in comparison with Con group (Figs 4A and 7A), indicating that our *in vitro* IR model was successful. Met decreased the generation of ROS during IR process (Fig 7A), and up-regulated expressions of MnSOD and catalase (Fig 7B and 7C), indicating that Met protected heart from IR injury by up-regulation of endogenous antioxidant enzymes.

Excessive generation of ROS is a main reason for apoptosis of myocardiocytes [6]. Lower expressions of apoptotic proteins Bax and cleaved caspase-3 with decreased apoptotic cells were found In IR+Met group *ex vivo* (Fig 3C and 3D). Additionally, Met treatment significantly inhibited ROS-mediated apoptosis (Figs 5 and 6) and improved mitochondrial ΔΨm (Fig 6A) *in vitro*. Zheng et al. [28] reported that Met inhibited apoptosis in the diabetic retinas via LKB1/AMPK/ROS signaling pathway. Does Met inhibit apoptosis during myocardial IR via the same signaling pathway? We found that as an inhibitor of AMPK, Compound C markedly decreased the expression of AMPK (Fig 4B), depleted the positive effect of Met on reduction of ROS and up-regulation of antioxidant system (Fig 7), then abolished the anti-apoptotic effect of Met (Figs 5 and 6), indicating that Met inhibited apoptosis depended on AMPK/ROS signaling pathway. Although we have proved that metformin could attenuate myocardial ischemia reperfusion injury via up-regulation of antioxidant enzymes in rat both *in vitro* and *ex vivo*, limitation of this study is lack of *in vivo* myocardial model and this will be performed in the future studies.

## Conclusion

Taken together, we demonstrated that the cardioprotective effect of Met depended on AMPK/ antioxidant enzymes signaling pathway. To our knowledge, this is the first time that the relationship between Met treatment and AMPK/ antioxidant enzymes signaling pathway during myocardial IR injury is explained. Therefore, Met can be used as a potential cardioprotective adjuvant in myocardial IR therapy and the up-regulation of AMPK/ antioxidant enzymes signaling pathway will be a promising modality for clinical myocardial IR therapy.

## Supporting information

S1 File NC3Rs ARRIVE guidelines checklist 2014. (DOCX)
