# Supplementary material for: Metformin attenuates myocardial ischemia-reperfusion injury via up-regulation of antioxidant enzymes
Source: PLoS One. 2017 Aug 17;12(8):e0182777. doi: 10.1371/journal.pone.0182777 (PMC5560646; doi:10.1371/journal.pone.0182777)

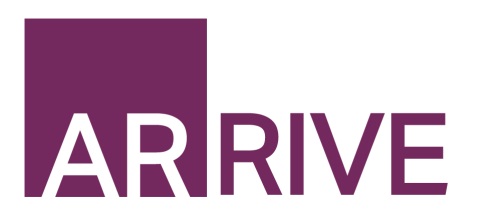


The ARRIVE Guidelines Checklist

Animal Research: Reporting In Vivo Experiments

Carol Kilkenny^1^, William J Browne^2^, Innes C Cuthill^3^, Michael Emerson^4^ and Douglas G Altman^5^

*^1^The National Centre for the Replacement, Refinement and Reduction of Animals in Research, London, UK, ^2^School of Veterinary Science, University of Bristol, Bristol, UK, ^3^School of Biological Sciences, University of Bristol, Bristol, UK, ^4^National Heart and Lung Institute, Imperial College London, UK, ^5^Centre for Statistics in Medicine, University of Oxford, Oxford, UK.*

|  | | ITEM | RECOMMENDATION | Section/ Paragraph |
| --- | --- | --- | --- | --- |
| 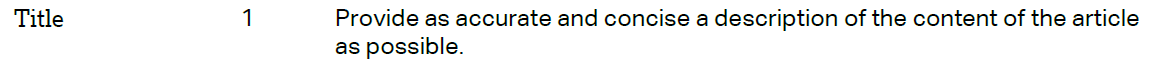 | | | Title page |  |
| 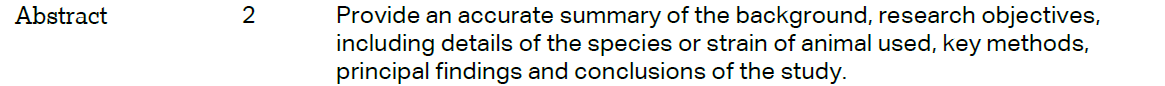 | | | Abstract/ paragraph 1 |  |
| INTRODUCTION | | |  |  |
| 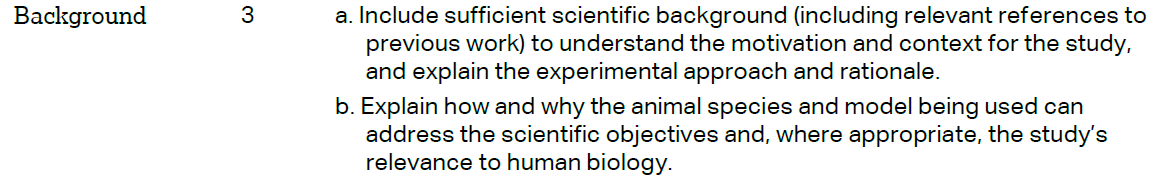 | | | Introduction/ paragraph 2-4 |  |
| 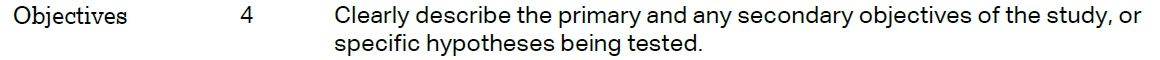 | | | paragraph 5 |  |
| METHODS | | |  |  |
| 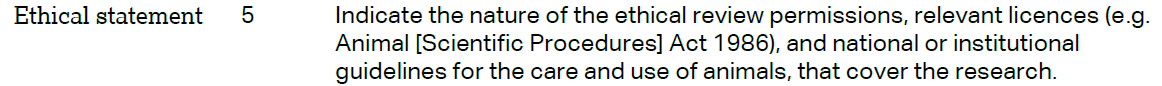 | | | Materials and Method/  paragraph 6 |  |
| 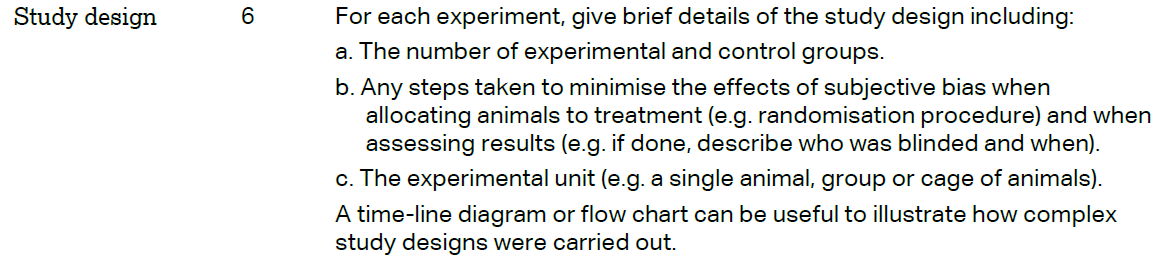 | | | Materials and Method/  paragraph 7 |  |
| 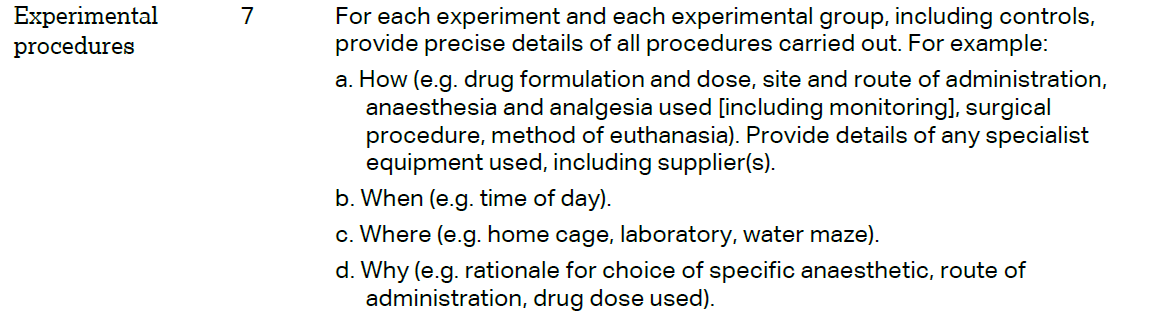 | | | Materials and Method/  paragraph 7 |  |
| 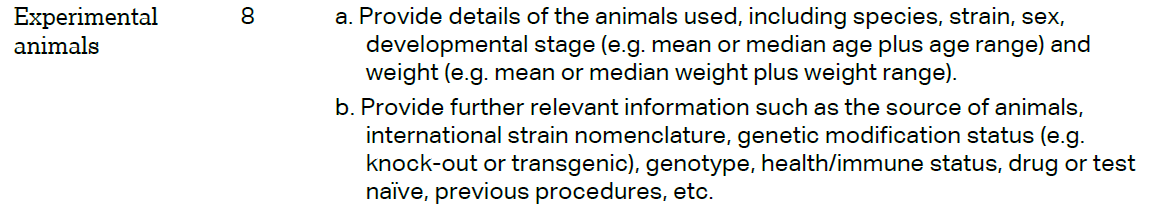 | | | Materials and Method/  paragraph 7 |  |

The ARRIVE guidelines. Originally published in *PLoS Biology*, June 2010^1^

| 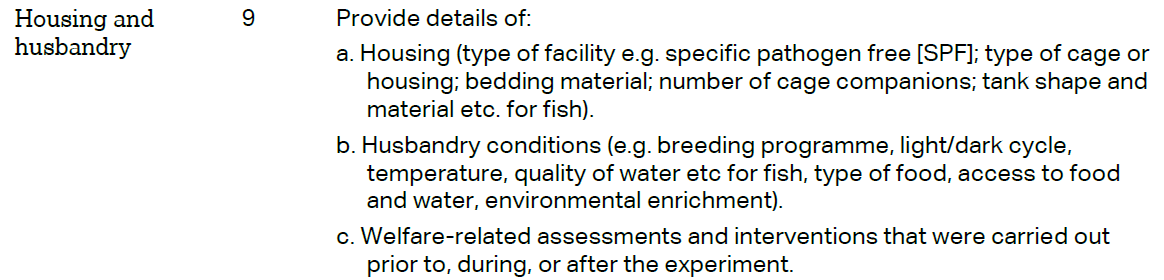 | Materials and Method/  paragraph 6 | |
| --- | --- | --- |
| 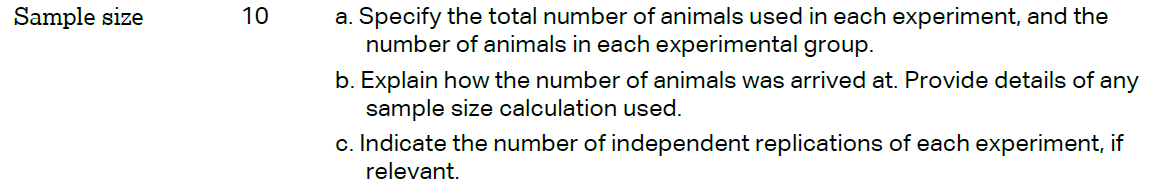 | Materials and Method/  paragraph 7 | |
| 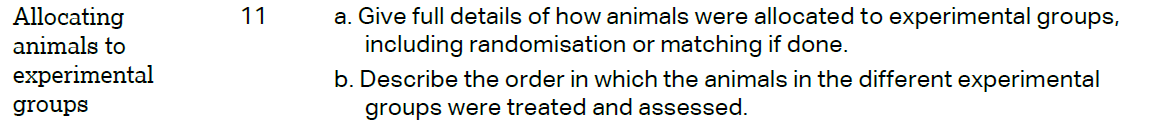 | Materials and Method/  paragraph 7 | |
| 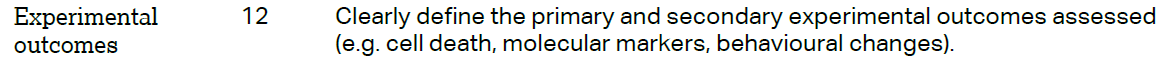 | Materials and Method/  paragraph 8-16 | |
| 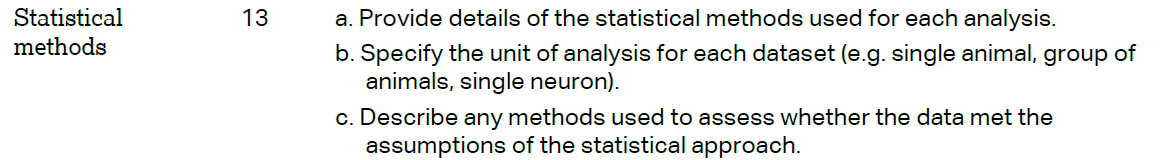 | Materials and Method/  paragraph 17 | |
| RESULTS |  | |
| 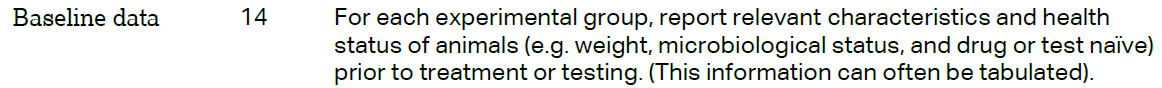 | Results and discussion/ | |
| 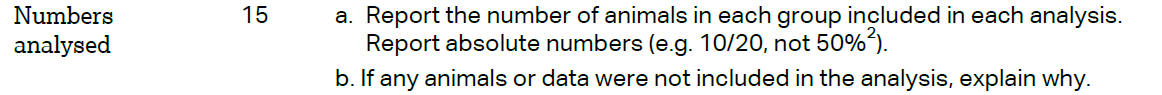 | Materials and Method/ paragraph 7 | |
| 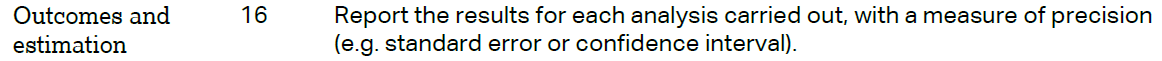 | Results and discussion/  paragraph 18-25 | |
| 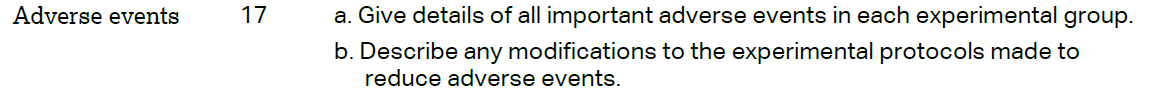 | Materials and Methods paragraph 7 | |
| DISCUSSION |  | |
| 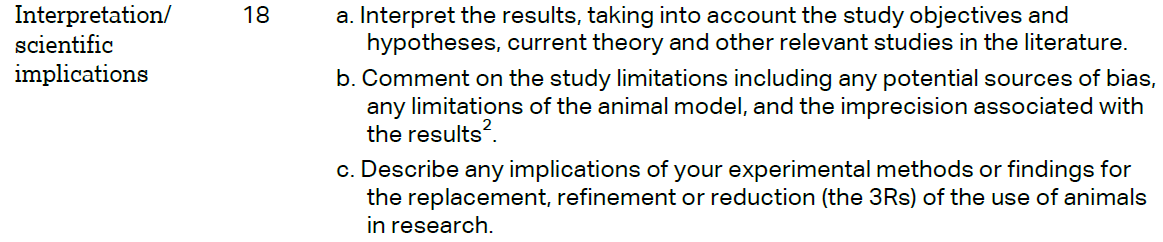 | Results and discussion/  paragraph 26-29 | |
| 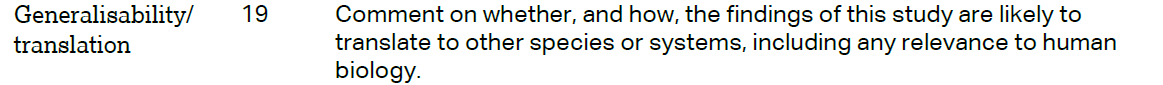 | Conclusion/  Paragraph 30 | |
| 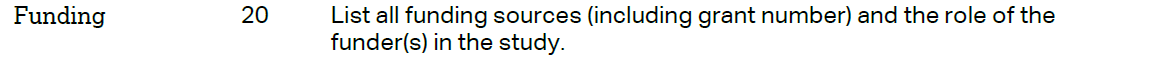 | | Funding Statement/  Paragraph 32 |


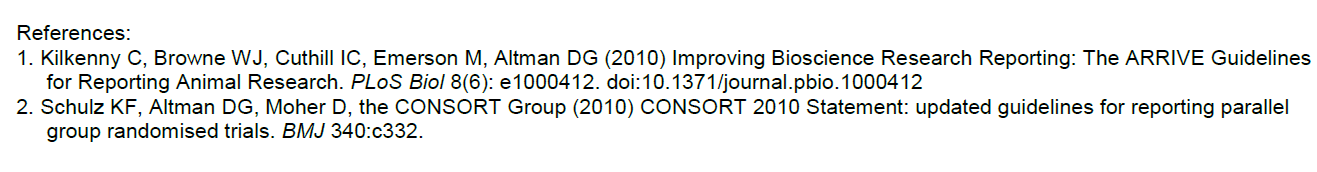

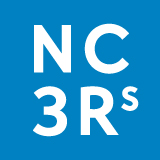

Supplement: S1 File — (DOCX) [file pone.0182777.s001.docx]
